# Homœopathy in the University of Michigan

**Published:** 1875-12

**Authors:** V. H. Taliaferro

**Affiliations:** Dean of Faculty


Homœopathy in the University of Michigan.—The faculty of the University of Michigan certainly occupy at present a very unenviable position before the profession. Let them put it as they may, if Prof. Sager's statement is correct, they stand convicted of not only not objecting to the union or co-partnership arrangement, but inviting it. If this statement is true, and we have waited long and anxiously to see it disproved, the original bill before the Legislature, and which passed the Senate, did not contemplate this unholy union, but proposed to appropriate six thousand dollars to establish a "complete college of homœopathy," to be located, not at Ann Arbor, but at any city or village, except Ann Arbor, in the State, making the most liberal appropriation. But here is the statement of Dr. Sager, who was at that time a member of the faculty :

"It will be recollected that about the middle of the session of the last Legislature, a bill was introduced into the Senate, drawn up by one of the most prominent homœopathic doctors, asking an appropriation of six thousand dollars for the establishment of a complete college of homœopathy in such city or village of the State as would contribute most liberally to the expenses involved in such location, Ann Arbor being excepted. In this form the bill passed the Senate with little opposition.

"Within a day or two after the introduction of this bill, and prior to its passage in the Senate, a meeting of the medical faculty was called by two of the oldest members, at which they proposed that the faculty should intimate a willingness to make some concession to the homœopaths by which they might be allowed to acquire the long-sought-for connection with the University. This proposition was negatived by the majority of the faculty, chiefly on the ground that the circumstance called for no concession. Near the close of the session, the bill passed the House in a modified form, authorizing the Board of Regents to locate the new school at Ann Arbor. The faculty were again convened, and requested to intimate an acceptance of the act of the Legislature, but hesitated to express any definite opinion. The member of the faculty at whose request the faculty had been convened went immediately to Lansing, to co-operate with a member of the Board of Regents to procure the passage of some appropriation bills for the hospital and college of dentistry, which, from opposition of the homœopaths, had previously failed. A public announcement was made by a Regent that the board would accept the appropriation and carry out the law. Whereupon the desired appropriations were promptly made, and assurance privately given by the friends of homœopathy in the Legislature that any other appropriation would be made if the medical faculty would cease to offer opposition.

"A few weeks later the faculty were convened by the request of Regent Rynd, and his plan of organization of the homœopathic school was submitted to them. Every member of the teaching faculty but one being present, it received their assent. Thus indorsed by the teaching faculty, it was soon after adopted by the Board of Regents. The minutes of the board contain not the slightest trace of any dissent on the part of the faculty, not the least intimation of any protest."

In reading the above extracts from this damaging pamphlet of Dr. Sager's, we have been irresistibly impressed with the idea that the change in the bill and the union of the two schools was the result of the manifestation of members of the faculty of the University.

Until this statement of Dr. S. is successfully disproved, the faculty stand convicted of tampering with the unclean thing.

Doctor Bruce's paper on "Malarial Hematuria," to be found in the original department of this number of the Journal, was read before the South Georgia Medical Association, at its meeting in October, and its publication requested by that body. From an oversight, the proper credits were omitted.

In this connection we will state, for the information of our readers in that section of the State, that the third meeting of this association will be held in Albany, on the second Tuesday in December. While this is a local association, and composed principally of physicians of the second Congressional district, still members of the profession in other portions of the State are cordially invited to be present.

We learn that a number of physicians, not yet members of the association, will be in attendance, and that the meeting will be of unusual interest. We hope to be present.

---

I Have Been Informed by some of the graduates of the Atlanta Medical College that their names have been omitted publication in the annual announcement of the institution. This I regret very much, as it was entirely unintentional on my part, and I would be glad if others, whose names have been omitted, would inform me of the fact. I will take pleasure in correcting all such omissions in every future announcement.

V. H. Taliaferro, M.D.,
*Dean of Faculty.*

